# Impact of Ground Granulated Blast Furnace Slag on Calcium Leaching of Low-Heat Portland Cement Paste

**DOI:** 10.3390/ma17153857

**Published:** 2024-08-04

**Authors:** Chunmeng Jiang, Li Xia, Shuangxi Li, Xiaoqing Li, Yingjie Chen, Jian Liu

**Affiliations:** 1 College of Hydraulic and Civil Engineering, Xinjiang Agricultural University, Urumqi 830052, China; xiali0914@163.com (L.X.); xjlisx123@xjau.edu.cn (S.L.); lixq_xj@163.com (X.L.); chenyj2021@xjau.edu.cn (Y.C.); xjliujian117@163.com (J.L.); 2 Xinjiang Key Laboratory of Hydraulic Engineering Security and Water Disasters Prevention, Urumqi 830052, China

**Keywords:** calcium leaching, low-heat Portland cement, ground granulated blast furnace slag, mechanical property, damage mechanism

## Abstract

Low-heat Portland cement and ground granulated blast furnace slag are widely used for the preparation of hydraulic concrete. Nevertheless, the effect and mechanism of corrosion on low-heat Portland cement paste mixed with ground granulated blast furnace slag need to be further explored. This paper investigated the impact of ground granulated blast furnace slag on the calcium leaching of low-heat Portland cement paste by evaluating its mass loss, porosity, leaching depth, compressive strength, and Vickers hardness, and comparing it with the leaching performance of ordinary Portland cement paste. Furthermore, the phase composition and morphology of low-heat Portland cement paste containing ground granulated blast furnace slag were analyzed by X-ray diffraction, mercury intrusion porosimetry, and scanning electron microscopy. The results indicate that, after 180 days of soaking in ammonium chloride solution, the mass loss rate, growth rate of porosity, leaching depth, and compressive strength loss rate of low-heat Portland cement paste were 8.0%, 43.6%, 9.1 mm, and 27.7%, respectively, while those of ordinary Portland cement paste were 7.4%, 37.8%, 8.4 mm, and 30.1%, indicating that low-heat Portland cement paste is slightly more damaging than ordinary Portland cement. The addition of ground granulated blast furnace slag could significantly improve the leaching resistance of low-heat Portland cement. For instance, after adding 20% ground granulated blast furnace slag, the above test values were 2.4%, 28.5%, 5.6 mm, and 20.8%, respectively. The reason for this is that ground granulated blast furnace slag has the potential to reduce the porosity of low-heat Portland cement paste, and it can also undergo the secondary hydration reaction with its hydration product Ca(OH)_2_ to enhance the paste structure. Considering the cost performance, the suitable dosage of low-heat Portland cement paste for satisfactory leaching resistance is about 20%.

## 1. Introduction

Calcium leaching is a phenomenon in which the hydration products of concrete in long-term contact with environmental water are dissolved, and the calcium ions (Ca^2+^) diffuse from the cement paste to the surrounding water under concentration gradient or chemical erosion. This leads to an increase in porosity, a degradation of mechanical performance, and a modification of the microstructure, ultimately causing damage to the structure [1,2]. Due to its low hydration heat and high later strength of low-heat Portland cement (LHC) [3], it is widely used in mass concrete projects such as concrete dams, sluices, piers, and foundations. During service, hydraulic concrete is often completely or partially immersed in water, and different degrees of calcium leaching usually occur. Hence, the investigation of the calcium leaching resistance of LHC holds significant practical and theoretical significance.

For LHC, Xie [4] has provided a comprehensive overview of the existing literature on application standards, clinker production, performance development, and environmental assessment of LHC and/or LHC-based materials and found that the 7-day hydration heat of LHC is the most common and important indicator. Compared with ordinary Portland cement (OPC), it also has better crack resistance, a longer initial cracking time, and higher corrosion resistance. Cement-based materials are all spliced by the hydration products of cement [5], including C-S-H gel, Ca(OH)_2_, ettringite, etc. [6]. Ca(OH)_2_ is mainly composed of crystal shapes in the pores of materials, and Ca^2+^ ions are the most important element of the constituent part of the frame structure of C-S-H. With the dissolution of Ca^2+^ ions, Ca(OH)_2_ dissolves, and the calcium-rich type C-S-H gradually turns into the silicon-rich C-S-H [7,8], and the structure of cement-based materials is gradually destroyed. Codina [9] formulated and characterized low-alkalinity and low-heat cements, finding that the leaching test in pure water indicated a very slow decalcification of the samples. Therefore, several techniques, such as optical microscopy, SEM/BSE, X-ray microanalysis, and X-ray diffraction, were compared to estimate the degraded thickness. Jiang [10,11] measured the mechanical and physical properties of low-heat cement-based pastes exposed to ammonium chloride aqueous solutions. In addition to this, he also investigated the deterioration process of high belite cement pastes exposed to sulfate attack, calcium leaching, and the dual actions based on their comparative macro properties and microstructures.

Cement-based materials usually include cement paste and supplementary cementitious materials (SCMs), of which, fly ash and ground granulated blast furnace slag (GGBS) are the most widely used [12]. At present, many scholars have made plenty of achievements in their research on the mechanics and durability of fly ash-LHC-based materials. The study demonstrated that by incorporating a sufficient quantity of fly ash, the hydration heat of LHC-based materials would be further reduced, thereby enhancing the long-term mechanical deformation and durability of mass concrete produced with it [10,11,13,14]. In the corrosion environment, on the one hand, the incorporation of fly ash enhances the fluidity of cement paste, potentially improving its pore structure and reducing its porosity. On the other hand, it replaces part of the cement content and reduces the contents of Ca(OH)_2_. Furthermore, it exerts a pozzolanic effect, which is a chemical reaction between the active SiO_2_ or Al_2_O_3_ in SCMs and the cement hydration product, and reacts with Ca(OH)_2_ to form C-S-H gel [15], which alleviates the dissolution process.

GGBS is an ultra-fine powder produced by the quenching, drying, and grinding of molten particles discharged during the ironmaking process. Its chemical composition and hydration characteristics are similar to those of Portland cement, and its fineness is finer than that of cement particles. Moreover, it has the pozzolanic effect of mineral admixtures and the filling effect of microaggregates [16,17]. After incorporation into concrete, the compactness and corrosion resistance of concrete could be improved. Generally, the pozzolanic activity of GGBS is better than that of fly ash [18]. GGBS-LHC-based materials are widely used in various types of hydraulic mass concrete, but research on the long-term performance of their calcium leaching is rarely reported. In this research, the mass loss, porosity, leaching depth, compressive strength, and Vickers hardness of GGBS-LHC paste specimens were determined by an accelerated leaching test with ammonium chloride solution [19,20]. Furthermore, the mechanism of leaching damage was analyzed through mercury intrusion porosimetry (MIP), X-ray diffraction (XRD), and scanning electron microscopy (SEM). Additionally, the impact of GGBS on the leaching resistance of LHC paste was investigated accordingly.

## 2. Materials and Methods

### 2.1. Materials and Sample Preparation

The materials used in this experiment mainly include low-heat Portland cement (LHC, in Chinese Standard GB/T 200-2017 [21]. Jiahua Special Cement Co., Ltd., Leshan, China), P⋅O 42.5 ordinary Portland cement (OPC, in Chinese Standard GB 175-2007 [22]. Nanjing Conch Cement Co., Ltd., Nanjing, China), and GGBS (Henan Platinum Casting Materials Co., Ltd., Gongyi, China). Their chemical and mineral composition are illustrated in Table 1. Ammonium chloride (Tianjin Zhiyuan Chemical Reagent Co., Ltd., Tianjin, China) as a corrosion accelerating medium.

Three groups of LHC paste specimens were designed with GGBS mass substitution rates of 0%, 20%, and 40%, with a constant water–binder ratio of 0.4. OPC paste specimens were prepared as a control group. They were called OPC, LHC, L-20G, and L-40G. As per Table 2, cylindrical specimens of cement paste measuring Φ50 mm × 100 mm were poured, followed by immersion in saturated lime water, and cured at an ambient temperature of 20 ± 2 ℃ for a duration of 90 days. After attaining the curing time, the specimens were removed and the surfaces were thoroughly dried. After that, the two ends of the specimens were sealed with epoxy resin. Afterwards, all of the cement samples were placed in a solution of NH_4_Cl at 6 mol/L to test their leaching performance. In order to maintain the concentration of the solution and prevent the carbonization of the specimens, it was necessary to replace the NH_4_Cl solution every 30 days.

### 2.2. Test Methods

**Figure 1:**
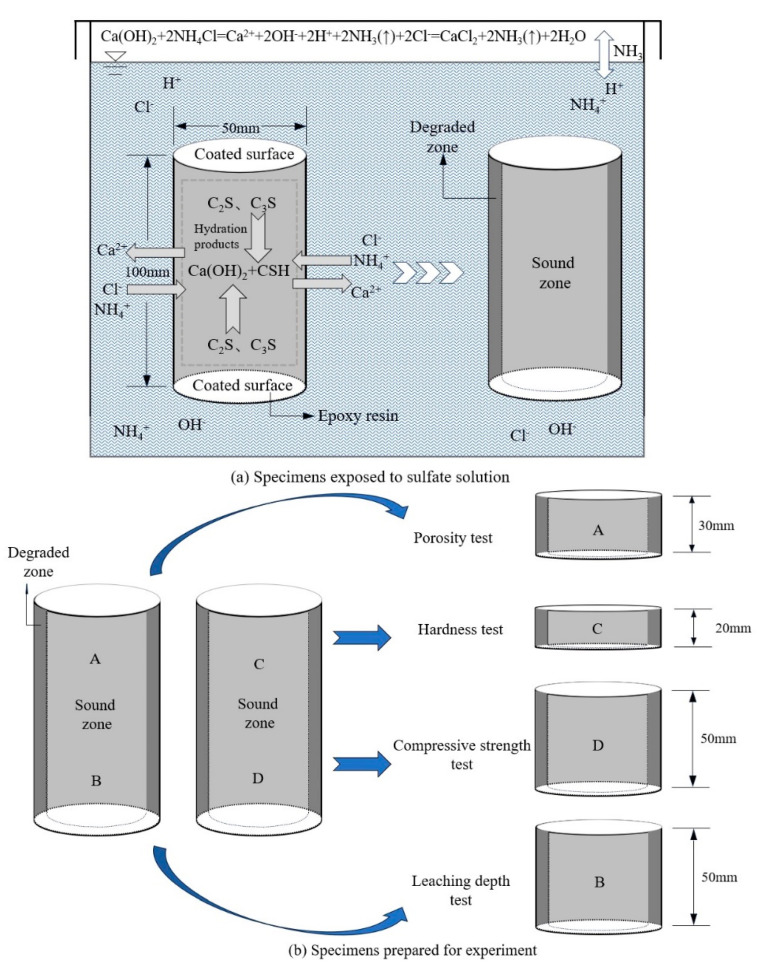
Scheme of exposure condition and testing. Note: The arrows indicate gas.

The cement paste specimens with a duration of 14 days, 28 days, 56 days, 91 days, 140 days, 180 days, and 270 days were removed from the NH_4_Cl solution. The tests were carried out according to Figure 1, and the specimens were cut into small cylinder specimens of different sizes for testing. Specimen A with a size of Φ50 mm × 30 mm was used for the porosity test, specimen B with a size of Φ50 mm × 50 mm was used for leaching depth measurement, specimen C with a size of Φ50 mm × 20 mm was used for the Vickers hardness test, and specimen D with a size of Φ50 mm × 50 mm was used for the compressive strength test. The final data were calculated as an average of the three tests prepared for each test.

#### 2.2.1. Mass Loss and Porosity

Mass loss and porosity are common indicators of the Ca^2+^ leaching process [23]. The mass change of the specimens before and after corrosion was measured by a high-precision electronic balance. The mass loss rate (*W*) and porosity (*P*) were calculated by Equations (1) and (2).

(1) $$W=\frac{G_{0}-G_{1}}{G_{0}}\times 100\%$$

(2) $$P=\frac{m_{1}-m_{0}}{V}\times 100\%$$

where *G*_0_ and *G*_1_ are the weights of cement paste specimens before and after leaching, which are dried to a normal value in the laboratory before measurement, g; *m*_1_ is the dry mass of the saturated surface of the specimen, g; *m*_0_ is the drying mass of the specimen after drying at 105 °C for 24 h, g; *V* is the volume, cm^3^.

#### 2.2.2. Leaching Depth

The cement paste specimens were taken out and cut along the diameter direction with a cutting machine at the time of arrival. The sections were immediately dried after being washed with clear water. To determine the leaching depth, an ethanol–phenolphthalein solution at a concentration of 1% was sprayed onto the cross-sections of the specimens. Under the magnifying glass, the leaching depth of specimens in each group was measured using a vernier caliper, and the experimental data were taken as the average of the depth of 8 measuring points.

#### 2.2.3. Compressive Strength

The compressive strength test is the most common test method for cementitious materials, and the measured results reflect the most basic and important mechanical properties of the material and are also a vital index to characterize the degree of deterioration of the specimens [24]. The specimens were placed on a CSS-44100 Electronic Universal Testing Machine (Suzhou Zhongse Chinese Copper Co., Ltd., Suzhou, China) for the test, and the loading rate was set to 0.1 mm/min. The failure load was recorded, and the compressive strength ($\sigma_{t}$) and strength loss ($\Delta \sigma_{t}$) of the leached specimens were calculated as Equations (3) and (4).

(3) $$\sigma_{t}=\frac{F_{t}}{A}$$

(4) $$\Delta \sigma_{t}=\frac{\sigma_{0}-\sigma_{t}}{\sigma_{0}}\times 100\%$$

where *F_t_* is the failure load, KN; *A* is the cross-sectional area of the cylindrical paste specimen, m^2^; $\sigma_{0}$ and $\sigma_{t}$ are the strengths of initial cylinder specimens and leached samples respectively, MPa.

#### 2.2.4. Vickers Hardness

Vickers hardness of a material is its resistance to external pressure on its surface, which can reflect the microstructure changes of the material and has locality and immediateness [25]. The surfaces of the specimens with size of Φ50 mm × 20 mm after cutting were polished with sandpaper and then placed on the HDX-1000TC microhardness tester. The test load was set to 0.981 N, the holding period was 15 s, and the Vickers hardness was measured every 2.5 mm from the surfaces to the interior along the erosion direction. The hardness results at the same position were taken from the average of the 8 points, and the aggregate should be avoided during the test.

#### 2.2.5. Mercury Intrusion Porosimetry

The methodology of MIP identifies the distribution of pore sizes within cement-based materials [26]. Its essence is to extract the gas in the connected pores inside the porous material, fill the pores with mercury under the action of external pressure, and calculate the pore-related parameters by the amount of mercury pressed. The instrument used for the MIP test is AutoPore Iv 9510 (Micromeritics, Norcross, GA, USA). The cement paste specimens of LHC and L-40G prior to dissolution and 91 days of leaching duration were subjected to mercury intrusion testing, and the modification in pore structure revealed the deterioration of the paste’s microstructure. The samples of different durations used in the test were taken from the slurry within 0.5 cm from the surface corrosion surface along the corrosion direction according to the unified standard.

#### 2.2.6. X-ray Diffraction

XRD was used to determine the crystal structure of the materials using the diffraction principle and carry out more accurate phase analysis [27]. The instrument used for XRD measurements is D8 Advance (Bruker, Berlin, Germany). XRD analysis was performed on the surface samples of OPC, LHC, and L-40G pastes before dissolution and 91 days of leaching duration, and the changes in the composition of the specimens before and after corrosion were compared. The specimens to be tested at the duration of 91 days in each group were taken out, and the small cements in the corrosion damage area were cut and ground to a square hole sieve capable of passing 0.08 mm, and then the cement powder was placed in a vacuum drying oven at 38 ℃ for 24 h, followed by XRD.

#### 2.2.7. Scanning Electron Microscope

SEM can observe the particle morphology and structural characteristics of hydration products in hardened pastes of cementitious materials at different leaching durations. Hitachi SU8010 (Hitachi, Tokyo, Japan) was used as the SEM instrument. The surface samples of OPC, LHC, and L-40G pastes prior to dissolution and after 91 days of leaching duration were collected for SEM observation of the microstructure and structure. The specimens of each group that reached the duration were submerged in anhydrous ethanol for a duration of 24 h, followed by removal, drying, and breaking. For 30 min, the fragments in the corrosion damage area were dried naturally. After gold plating the surfaces, SEM was conducted under high vacuum conditions.

## 3. Results and Analysis

### 3.1. Mass Loss

**Figure 2:**
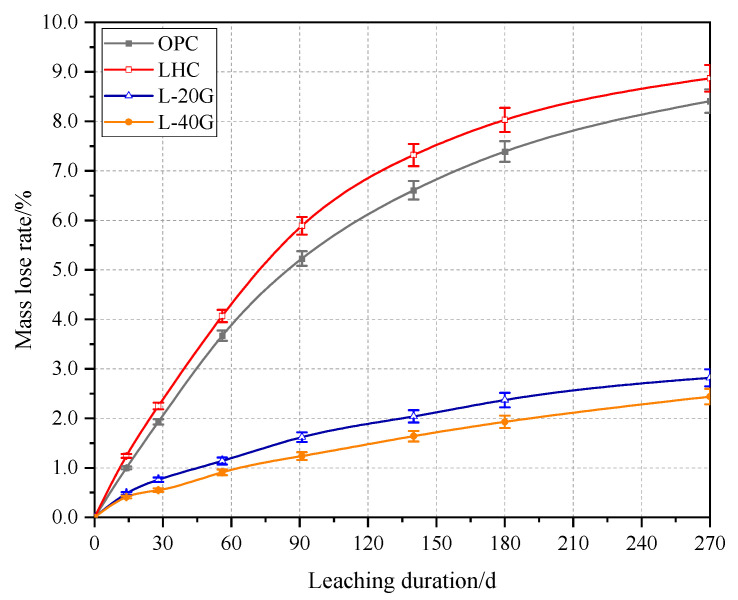
Mass loss rate of cement paste specimens.

The mass loss rates of OPC, LHC, L-20G, and L-40G pastes with different leaching durations were calculated, and the results are shown in Figure 2. It can be seen from the figure that the mass loss rates of the four groups of cement paste specimens increase with the extension of the leaching duration and grow at a higher rate in the initial meltdown than in the latter. The mass loss rates of OPC and LHC pastes are 5.2% and 5.9% when exposed for 91 days, and 7.4% and 8.0% when exposed for 180 days, respectively. Depending on the sample’s mass loss, OPC’s leaching resistance is slightly better than that of LHC during the test period. The mass loss rates of L-20G and L-40G pastes at 91 days of leaching were 1.6% and 1.2%, and the mass loss rates at 180 days of leaching were 2.4% and 1.9%, indicating that the LHC with 20% and 40% GGBS had better corrosion resistance than OPC.

The corrosion resistance of LHC is slightly worse than that of OPC. According to the chemical composition analysis of the two kinds of cement in Table 1, it can be seen that the initial calcium–silicon ratio of LHC is relatively low. Even though the amount of Ca(OH)_2_ produced by the hydration of LHC is small, with the dissolution of Ca^2+^ ions, the macroscopic physical and mechanical properties of cement paste are significantly lower than those of OPC. GGBS’s incorporation, on the one hand, exerts a morphological influence, enhancing the pore structure of cement paste and reducing porosity. On the other hand, it reduces the amount of clinker mineral composition in cement paste. Simultaneously, owing to its high pozzolanic activity, it can be secondary hydrated with Ca(OH)_2_ in cement hydration products, thereby reducing the yield of Ca(OH)_2_, reducing the likelihood of encountering ammonium ions, and allowing Ca(OH)_2_ to diffuse into the paste, thereby alleviating the dissolution of Ca^2+^ ions. Nevertheless, the mass loss rates of the three types of LHC pastes are not directly proportional to the quantity of GGBS. When the content of GGBS is increased from 20% to 40%, the corrosion resistance of LHC does not improve much compared with the dosage from 0% to 20%. From the perspective of cost performance, the suitable dosage is about 20%.

### 3.2. Porosity

**Figure 3:**
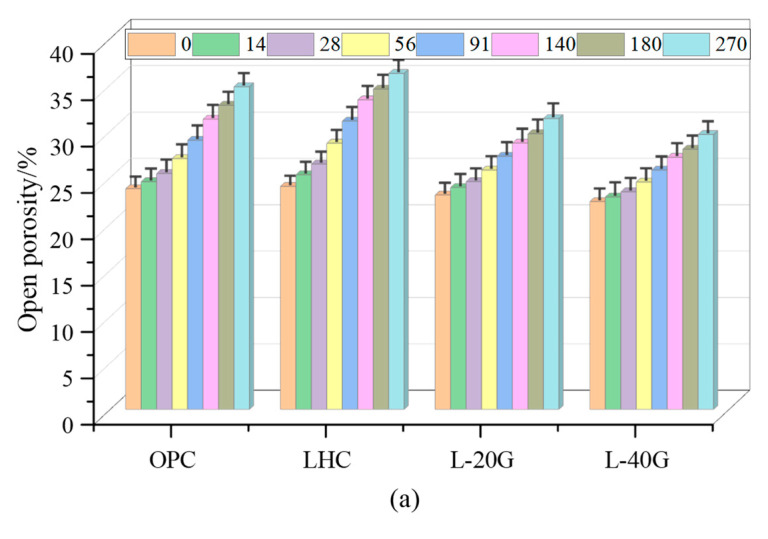

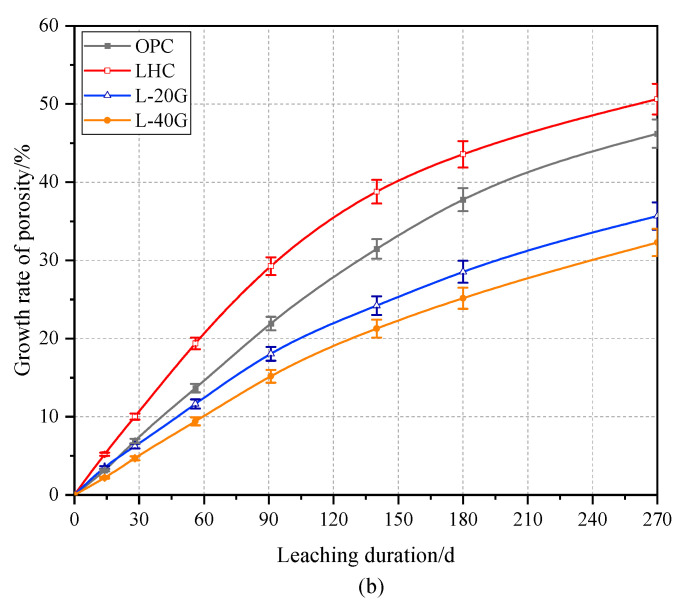
(**a**) Open porosity and (**b**) growth rate of porosity of cement paste specimens.

During different durations of leaching, the open porosities of OPC, LHC, L-20G, and L-40G pastes were evaluated, and the porosity growth rates were calculated. The results are shown in Figure 3. The four types of cement paste exhibit increased porosity upon prolonged immersion, and the rate at which they become porous increases rapidly upon the onset of corrosion. After 270 days, the rising slope tends to be gentle, consistent with the development law of its mass loss rate curves.

The porosity growth rates of OPC and LHC pastes were, respectively, 21.9% and 29.3% over 91 days of corrosion and 37.8% and 43.6% over 180 days of corrosion, indicating that OPC’s leaching resistance was slightly superior to that of LHC during the testing period. The porosity growth rates of L-20G and L-40G pastes at 91 days of dissolution were 18.1% and 15.2%, whereas the porosity growth rates at 180 days of dissolution were 28.5% and 25%. Thus, its leaching resistance surpasses that of OPC. Additionally, these phenomena are consistent with mass loss rates and the decrease in porosity growth rates of LHC paste, which are not proportional to the content of GGBS.

### 3.3. Leaching Depth

In a soft water or acidic solution environment, the dissolution of Ca^2+^ ions in cement-based materials is primarily affected by the diffusion mechanism [28]. According to existing research, Fick’s law can characterize the relationship between the leaching depth of concrete materials and the square root of the leaching duration, as shown in Equation (5) [10].

(5) $$d_{t}=k\cdot \sqrt{t}$$

where *d_t_* is the leaching depth, mm; *k* is the coefficient related to the raw material and mix proportion; and *t* is the leaching duration, days.

**Figure 4:**
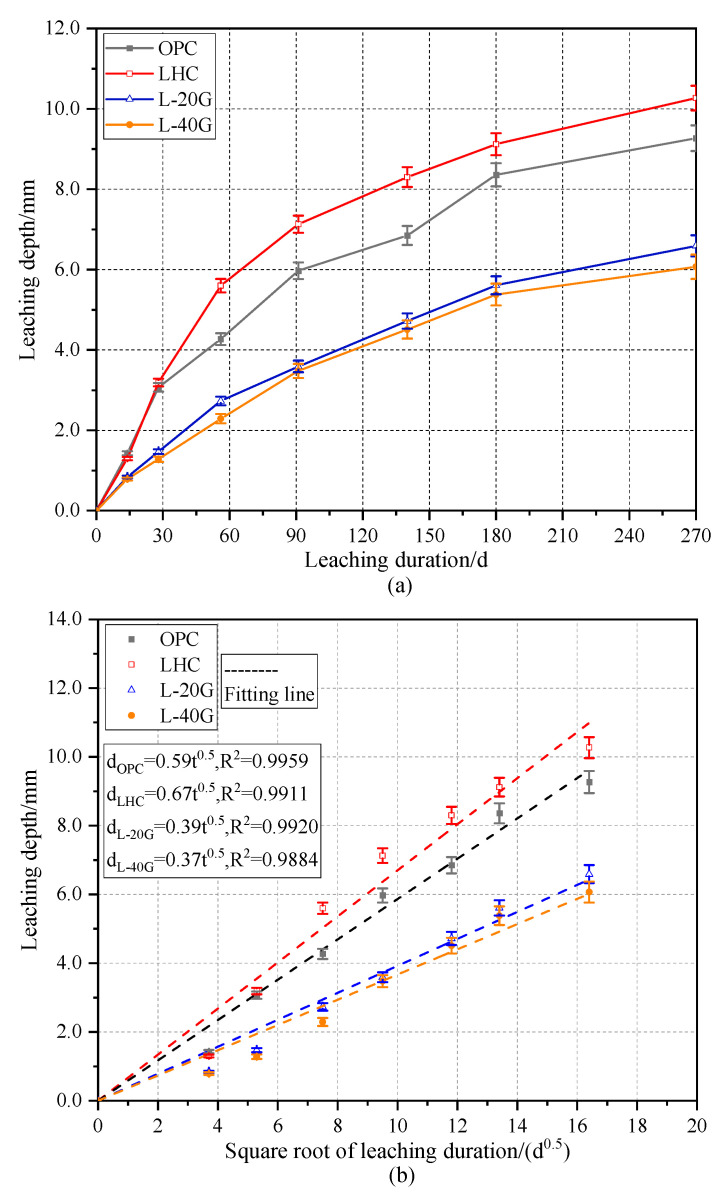
(**a**) Leaching depth and (**b**) fitting line of leaching depth of cement paste specimens.

The leaching depths of OPC, LHC, L-20G, and L-40G pastes were tested, and the data were fitted according to Formula (5). The results are shown in Figure 4. The leaching depths of the four cement pastes increased as the soaking time goes on, and the correlation coefficients were high, which were all about 0.99, indicating the rationality of using Fick’s law to characterize. The leaching depth of LHC paste was slightly deeper than that of OPC paste, and the depths of L-20G and L-40G pastes were lower than those of LHC paste. This indicates that the corrosion resistance of OPC is slightly better than that of LHC, and the corrosion resistance of LHC with GGBS is stronger than that of OPC. The leaching depths of GGBS-LHC paste specimens decreased with the increase in GGBS content, and the depths of 20% and 40% GGBS were close, which is consistent with the relative size of its porosity.

### 3.4. Compressive Strength

**Figure 5:**
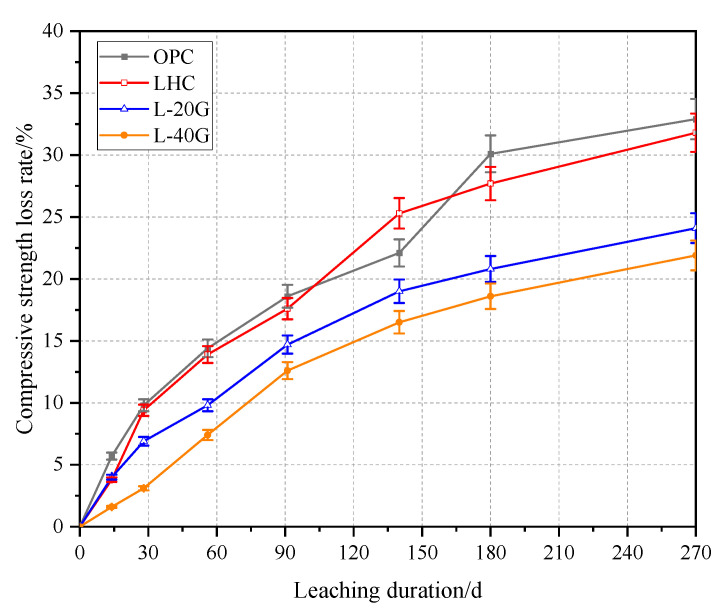
Compressive strength loss rate of cement paste specimens.

The strength loss rates of OPC, LHC, L-20G, and L-40G pastes with different leaching durations were calculated, respectively, and the results are shown in Figure 5. With the extension of the leaching duration, the strength loss rate curves of the four groups of cement paste specimens showed an upward trend, and the growth rates were faster in the early stage of dissolution and gradually slowed down in the later stage. The compressive strength of the cement specimens decreased with the occurrence of correlation. This is due to the continuous dissolution of Ca(OH)_2_ in cement pastes and the decalcification reaction of C-S-H gel, which results in the formation of loose and porous specimens, resulting in a continuous decrease in strength. The compressive strength loss rates of OPC and LHC pastes were similar, and the corresponding 91-day strength loss rates were 18.6% and 17.6%, respectively. The corresponding 180-day strength loss rates were 30.1% and 27.7%. The compressive strength loss rates of L-20G and L-40G pastes were lower than those of OPC. The 91-day strength losses were 14.7% and 12.6%, and the 180-day strength losses were 20.8% and 18.6%, demonstrating that the leaching resistance of LHC with GGBS is superior to that of OPC.

### 3.5. Vickers Hardness

**Figure 6:**
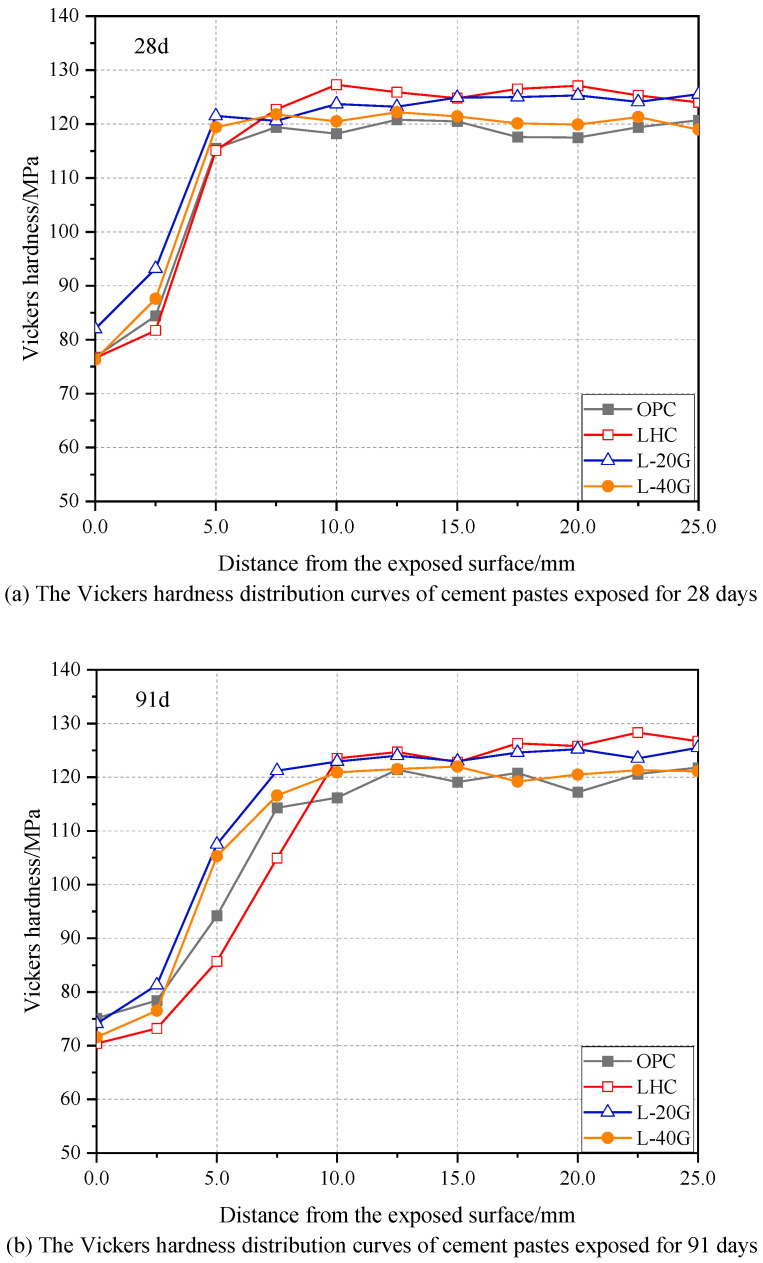

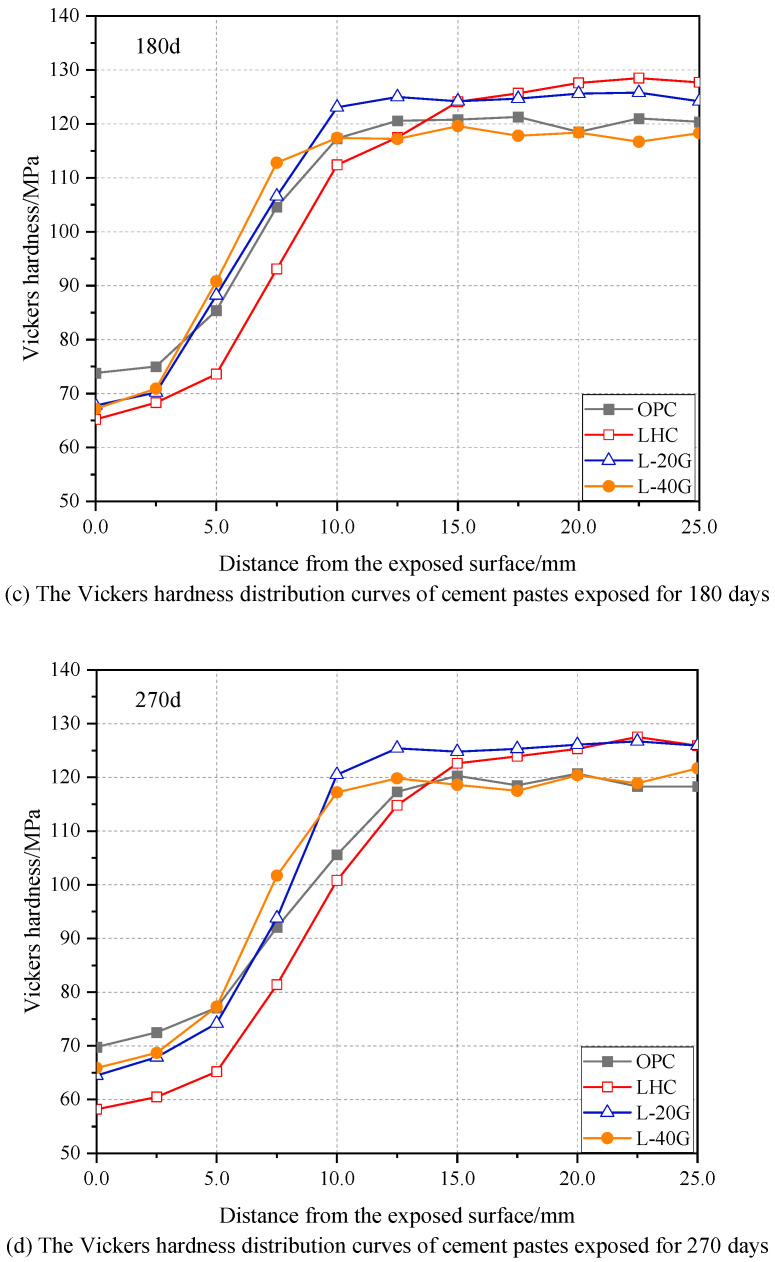
Vickers hardness of cement paste specimens.

The cross-section Vickers hardness distribution curves of OPC, LHC, L-20G, and L-40G pastes with different leaching durations were tested. The results are presented in Figure 6. According to the change trend, the erosion section can be divided into two parts: the corrosion area and the intact area. The Vickers hardness of LHC paste in the intact area is slightly larger than that of OPC, indicating that LHC’s hydration product structure is more compact and has higher initial mechanical properties. During the initial stage of leaching (28 d), the Vickers hardness curves of the deterioration area of OPC and LHC pastes exhibited similarity. However, with the extension of immersion age, the Vickers hardness distribution curve of LHC accelerated, leading to a gradual decrease in the hardness of the deterioration area compared with that of OPC. Furthermore, the decrease in depth of the sample corresponding to this region was also gradual, indicating that the mechanical properties of LHC paste under dissolution are less robust compared with OPC specimens.

The initial Vickers hardness of the L-20G was similar to that of LHC paste. When the content of GGBS increased to 40%, the initial Vickers hardness of the L-40G specimen was slightly lower than that of the LHC. The reason is that when the dosage is appropriate, the GGBS can produce secondary hydration with Ca(OH)_2_ in the cement pastes to further densify the structure, but when the dosage is high, the GGBS mainly plays a filling role, reducing the content of hydration products in the slurry, resulting in a decrease in mechanical properties. Moreover, the hardness values of the affected regions of the three groups of cement pastes exhibited a striking resemblance. The hardness values of L-20G and L-40G specimens were relatively close, both of them being greater than LHC, and the difference gradually increased with the extension of the leaching duration. It can be inferred that the incorporation of GGBS can effectively enhance the leaching resistance of LHC paste, and its suitable content is about 20%.

### 3.6. Mercury Intrusion Porosimetry

**Figure 7:**
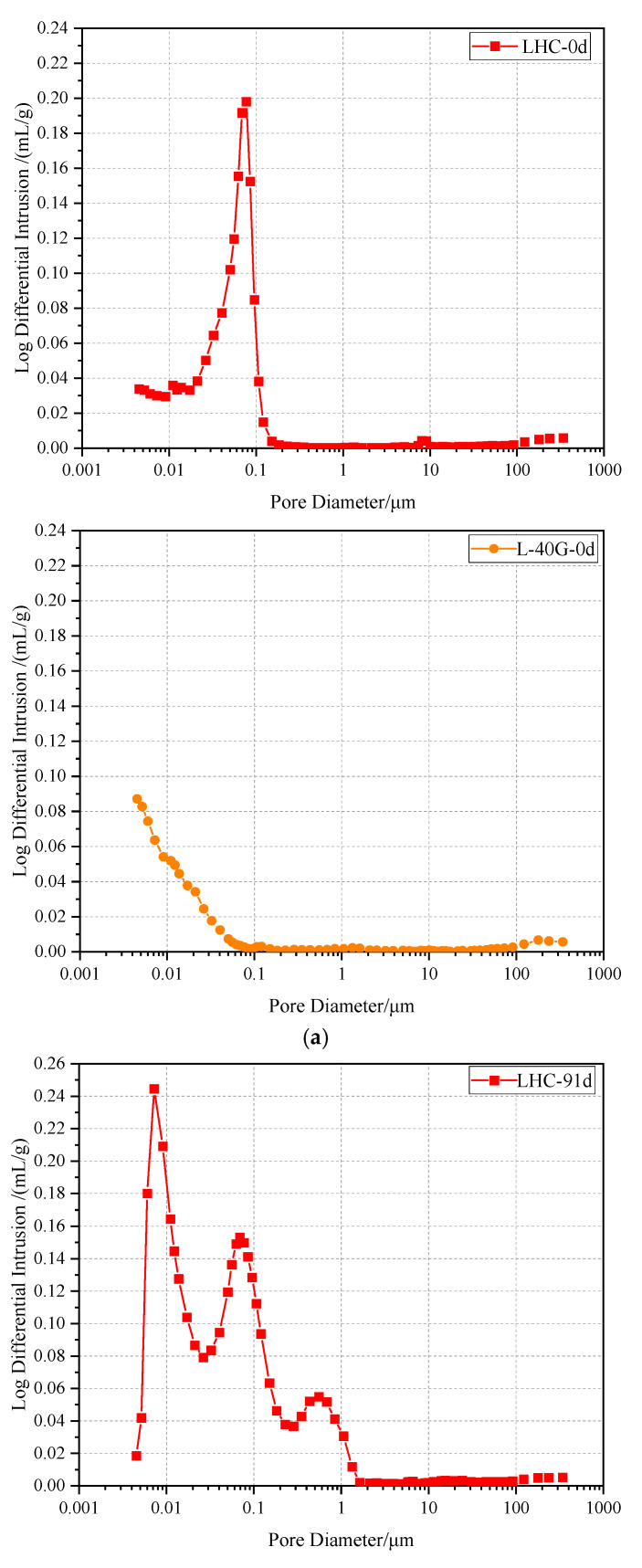

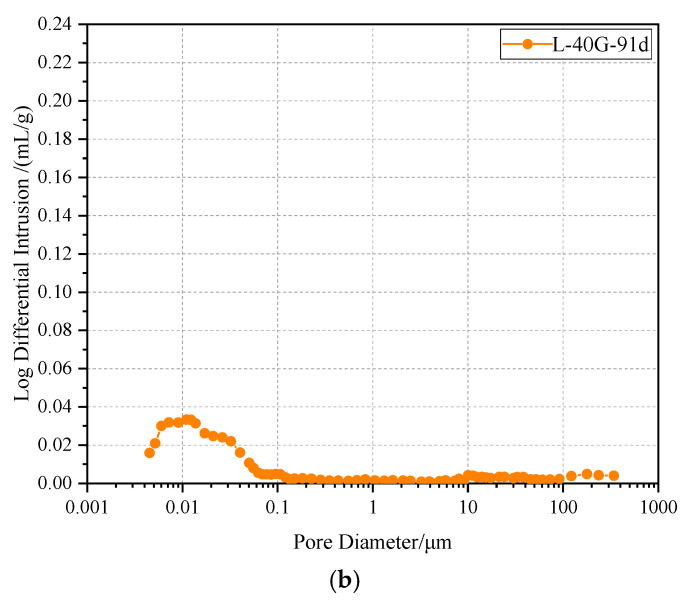
The cumulative mercury volume differential curve of the cement pastes (**a**) before leaching and (**b**) exposed for 91 days.

The leaching resistance of LHC and L-40G pastes was further tested by choosing for the mercury intrusion test before leaching and soaking in ammonium chloride solution for 91 days. The results are shown in Figure 7. It can be seen from the figure that the most probable pore sizes of the LHC paste specimen before and after 91 days of leaching were near 0.08 μm and 0.007 μm. The most probable pore diameters of the LHC paste specimen with 40% GGBS before leaching and exposed for 91 days were located near 0.005 μm and 0.01 μm. Before dissolution, the most probable pore size of L-40G was smaller (less than 0.01 μm) than that of the LHC specimen, and the number of pores was greatly reduced. The rationale behind this is that the GGBS exhibits a favorable filling effect, and concurrently, it undergoes hydration with the hydration product Ca(OH)_2_ to generate C-S-H gel, which effectively compacts the slurry structure to a certain extent. When the dissolution is 91 days, the most probable pore size of the GGBS-LHC specimen shifts to the right and the peak value decreases. The reason for this is that the slurry of the specimen is already dense at the commencement of dissolution, the number of slurry pores is minimal, and the dissolution process is sluggish. There is obvious hydration in the undissolved area of the specimen, and the dissolution is almost covered. The most probable pore size of the LHC specimen shifts to the left, the peak value increases, the pore size range expands, and the number of pores increases. The reason is that the corrosion phenomenon is gradually obvious, the hydration phenomenon is gradually covered up, and the pore structure of the specimen is seriously degraded. In summary, the GGBS has a good filling and pozzolanic effect, which is beneficial to the resistance of the slurry to corrosion at the initial stage of corrosion.

### 3.7. X-ray Diffraction

**Figure 8:**
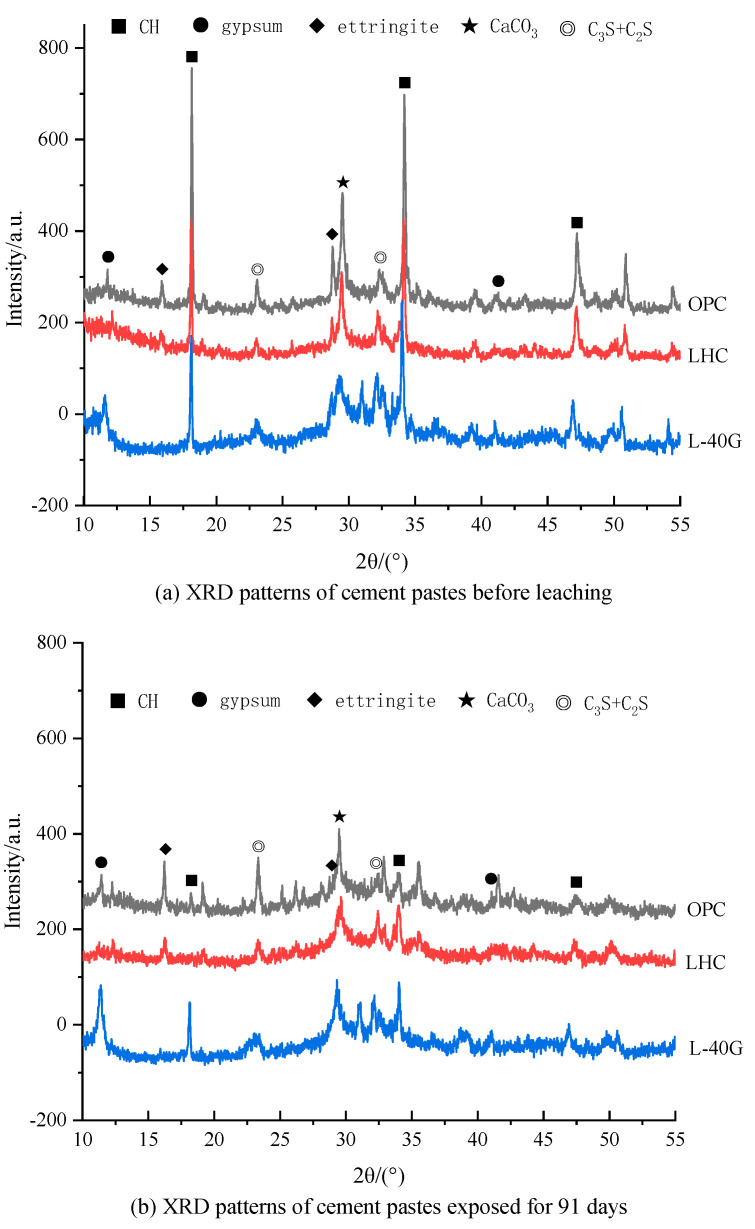
XRD patterns of cement pastes (**a**) before leaching and (**b**) exposed for 91 days.

Before and after 91 days of leaching, surface samples of OPC, LHC, and L-40G pastes were taken, and XRD analysis was carried out after grinding and drying treatment. The test results are shown in Figure 8. It can be seen from Figure 8a that the composition of the hydration products of the three groups of cement pastes was basically the same. The crystal phase diffraction peaks on the XRD pattern mainly included Ca(OH)_2_ (18.1°, 34.2°, 47.2°), unhydrated C_3_S and C_2_S minerals (23.1°, 32.4°), calcium carbonate (29.5°), gypsum (11.7°, 41.0°), and ettringite (15.9°, 28.9°). The relative contents of Ca(OH)_2_ in the three groups of cement pastes, from high to low, were OPC, LHC, and L-40G, which proves that the content of Ca(OH)_2_ generated by C_2_S hydration was lower than that of C_3_S. The incorporation of GGBS can reduce the content of clinker mineral composition in cement pastes, which leads to a decrease in Ca(OH)_2_ generated by hydration. Furthermore, there was no discernible disparity in the proportions of gypsum and ettringite present in the three cement pastes.

It can be seen from Figure 8b that the diffraction peak intensity of Ca(OH)_2_ in the XRD pattern of the three groups of cement paste specimens after 91 days of corrosion was significantly lower than that before leaching, but it still existed, indicating that the dissolution process at this stage was still dominated by the dissolution of Ca(OH)_2_. It is believed that the decalcification reaction of C-S-H gel was still in its initial stages or had not yet occurred. The peak intensity of Ca(OH)_2_ in the XRD patterns of OPC and LHC samples was relatively close, that is, the content of Ca(OH)_2_ in the surface layer of cement paste was basically the same after 91 days of corrosion, while the peak intensity of Ca(OH)_2_ corresponding to L-40G was significantly higher than that of LHC, which further confirms that the incorporation of GGBS can effectively reduce the dissolution rate of Ca^2+^ ions and improve the corrosion resistance.

### 3.8. Scanning Electron Microscope

**Figure 9:**
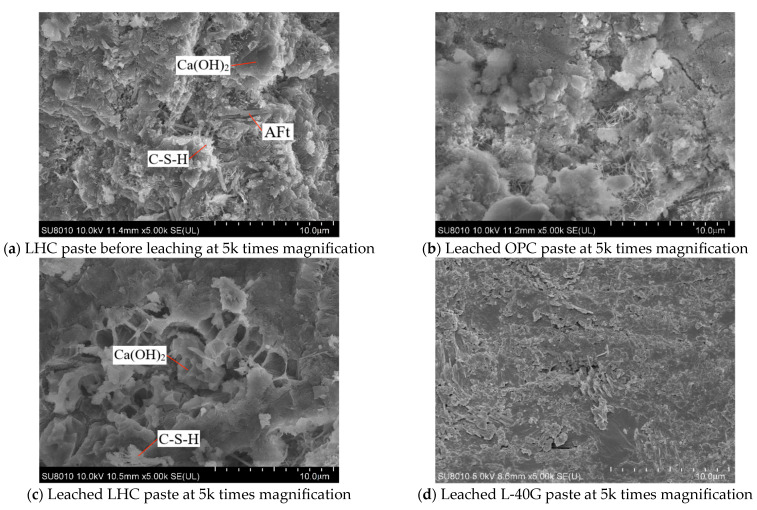
Morphology of L-40G, LHC, and OPC specimens.

The surface samples of LHC paste prior to corrosion and OPC, LHC, and L-40G pastes after 91 days of leaching were obtained for the purpose of conducting the SEM test to observe their microstructure modifications. The results are shown in Figure 9. It can be seen from Figure 9a,c that the cement paste skeleton before dissolution was flat and dense, while the cement paste skeleton after dissolution became loose and disordered, and its cement matrix structure presented a fishing net shape, indicating that Ca^2+^ ions have been dissolved in large quantities, resulting in a decrease in its structural compactness and an increase in porosity. For hydration products, the cement hydration products before dissolution had good morphology and dense structure, including needle-like ettringite (AFt), plate-like and hexagonal Ca(OH)_2_, and numerous flocculant C-S-H gels. However, the structure of the cement paste after dissolution was full of pores and holes, and it can be observed that the plate-like Ca(OH)_2_ edge had been damaged under dissolution, and the structural morphology of C-S-H gel had also changed to some extent. It is possible to infer from this that the decalcification reaction occurred.

It can be seen from Figure 9c,d that the structure of L-40G paste was relatively dense and the morphology of LHC was relatively poor. It has been speculated that the corrosion resistance of each group, ranging from strong to weak, is GGBS-LHC, OPC, and LHC. This suggests that the corrosion resistance of LHC is significantly enhanced following the addition of GGBS.

## 4. Conclusions

The deterioration laws of mass loss, porosity, leaching depth, compressive strength, and Vickers hardness of LHC paste specimens in an NH_4_Cl corrosion environment are similar to those of OPC, but there is a slightly higher degree of deterioration for the former compared with OPC. The main reason is that the initial Ca(OH)_2_ content of LHC paste was low, and when further dissolution occurred, its macro performance decreased more clearly than that of OPC. When the proportion of GGBS increased from 20% to 40%, the leaching resistance of the low-heat cement paste had not significantly improved. From the perspective of cost performance, the suitable content of GGBS is about 20%.

The macroscopic properties of GGBS-LHC paste specimens with different admixture contents were significantly better than those of LHC and OPC paste. Combined with the microscopic phase composition and morphology measured by MIP, XRD, and SEM, it can be inferred that due to the filling effect and pozzolanic effect of GGBS, the initial structure of the paste was densified. At the same time, GGBS reacts with Ca(OH)_2_ to form C-S-H gel with bonding properties, which further enhances corrosion resistance. Therefore, the addition of GGBS could significantly enhance the calcium leaching performance of LHC paste.

## Figures and Tables

**Table 1:** Chemical and mineral composition (%).

Material	CaO	SiO_2_	Al_2_O_3_	Fe_2_O_3_	MgO	SO_3_	R_2_O *	Mineral Composition			
								C_3_S	C_2_S	C_3_A	C_4_AF
LHC	58.74	22.82	3.55	4.28	4.99	2.43	0.39	28.7	43.9	2.1	13.0
OPC	62.83	20.50	5.61	3.84	1.70	3.07	1.05	48.0	22.9	8.4	11.7
GGBS	38.36	29.03	15.17	0.37	10.7	2.90	1.21	-	-	-	-

Note: * R_2_O = Na_2_O + 0.658K_2_O.

**Table 2:** Mixed proportions of cement pastes.

Sample Codes	P.LH/%	P.O/%	GGBS/%
OPC	0	100	0
LHC	100	0	0
L-20G	80	0	20
L-40G	60	0	40

## Data Availability

The original contributions presented in the study are included in the article, further inquiries can be directed to the corresponding author.
